# BASI74, a Virulence-Related sRNA in *Brucella abortus*

**DOI:** 10.3389/fmicb.2018.02173

**Published:** 2018-09-13

**Authors:** Hao Dong, Xiaowei Peng, Yufu Liu, Tonglei Wu, Xiaolei Wang, Yanyan De, Tao Han, Lin Yuan, Jiabo Ding, Chuanbin Wang, Qingmin Wu

**Affiliations:** ^1^China Animal Disease Control Center, Beijing, China; ^2^Department of Inspection Technology Research, China Institute of Veterinary Drug Control, Beijing, China; ^3^College of Veterinary Medicine, South China Agricultural University, Guangzhou, China; ^4^Key Laboratory of Preventive Veterinary Medicine of Hebei Province, Hebei Normal University of Science and Technology, Qinhuangdao, China; ^5^Institute of Animal Husbandry and Veterinary Medicine, Beijing Academy of Agriculture and Forestry Sciences, Beijing, China; ^6^Key Laboratory of Animal Epidemiology and Zoonosis, Ministry of Agriculture, College of Veterinary Medicine, China Agricultural University, Beijing, China

**Keywords:** *Brucella*, sRNA, virulence, post-transcriptional regulation, stress response, intracellular survival

## Abstract

*Brucella* spp. are intracellular pathogens that infect a wide variety of mammals including humans, posing threats to the livestock industry and human health in developing countries. A number of genes associated with the intracellular trafficking and multiplication have so far been identified in *Brucella* spp. However, the sophisticated post-transcriptional regulation and coordination of gene expression that enable *Brucella* spp. to adapt to changes in environment and to evade host cell defenses are not fully understood. Bacteria small RNAs (sRNAs) play a significant role in post-transcriptional regulation, which has already been confirmed in a number of bacteria but the role of sRNAs in *Brucella* remains elusive. In this study, we identified several different sRNAs in *Brucella* spp., and found that over-expression of a sRNA, tentatively termed BASI74, led to alternation in virulence of *Brucella* in macrophage infection model. The expression level of BASI74 increased while *Brucella abortus* 2308 was grown in acidic media. In addition, BASI74 affected the growth ratio of the *Brucella* cells in minimal media and iron limiting medium. Using a two-plasmid reporter system, we identified four genes as the target of BASI74. One target gene, BABI1154, was predicted to encode a cytosine-N4-specific DNA methyltransferase, which protects cellular DNA from the restriction endonuclease in *Brucella*. These results show that BASI74 plays an important role in *Brucella* survival in macrophage infection model, speculatively by its connection with stress response or impact on restriction-modification system. Our study promotes the understanding of *Brucella* sRNAs, as well as the mechanism by which sRNAs use to influence *Brucella* physiology and pathogenesis.

## Introduction

*Brucella* spp. as well as other bacteria are capable of quickly adapting to changing conditions to survive. Successful adaptation depends on changes in gene expression, which may take place at both transcriptional level and post-transcriptional level. Compared to a wide range of studies in transcriptional regulation, e.g., transcriptional regulators (Dong et al., 2013), two-component regulators (Abdou et al., 2013), quorum sensing systems (Brambila-Tapia and Perez-Rueda, 2014), only a limited number of studies focused on post-transcriptional regulation [especially small RNAs (sRNAs)] in *Brucella* spp.

Small RNAs usually have a length of 50–300 nt and most of them base-pair with mRNA and regulate mRNA stability or mRNA translation efficiency. According to the location of their genes on the chromosomes, sRNAs can be divided into two groups: (a) *cis*-acting sRNAs with the capacity of extensive base pairing, and (b) trans-encoded sRNAs, having limited potential of base pairing with the target mRNAs (Waters and Storz, 2009).

Previous studies have demonstrated that some sRNAs are involved in bacterial virulence in various pathogens (such as *Listeria, Salmonella, Vibrio*, and *Yersinia*). Two sRNAs (AbcR1 and AbcR2) regulating *Brucella* virulence were identified, and AbcR1 and AbcR2 double mutant was defective in both macrophage infection model and mice chronic infection model (Caswell et al., 2012; Sheehan and Caswell, 2017). One sRNA (BSR0602), which modulated *Brucella melitensis* intracellular survival was also reported (Wang et al., 2015). Based on the results of strand-specific RNA deep-sequencing approach, 1321 sRNAs were found in *B. melitensis* 16 M, and one sRNA, BSR0441, involved in bacterial virulence in both macrophages and mice infection models was also found (Zhong et al., 2016).

In previous studies, we integrated the output of two published sRNA detection programs (sipht and napp), and found a total of 129 sRNAs candidates, out of which 7 from 20 sRNA candidates were verified by RT-PCR (Dong et al., 2014). In this study, additional 43 sRNA from 109 remaining candidates were detected by RT-PCR and the role of all verified sRNAs in virulence of *Brucella* was examined by over-expression in the wild type strain *B. abortus* 2308. We identified and characterized one sRNA (BASI74) that significantly changed *Brucella* virulence in macrophage infection model.

## Materials and Methods

### Bacteria Strains and Culture Conditions

We performed a routine cultivation of *Escherichia coli* strains in Luria-Bertani (LB) broth or on LB agar plates with appropriate antibiotic supplementation, if necessary. The *Brucella* strains were routinely grown in tryptic soy broth (TSB, BD company) at 37°C or on tryptic soy agar medium incubated at 37°C under 5% CO_2_. Additionally, we added chloramphenicol (30 μg/mL), when we cultured the *Brucella* strains with chloramphenicol resistance. All of the bacterial strains were stored at -80°C and supplemented with 25% (v/v) glycerol. In order to determine the expression levels of the BASI74 under different conditions, we cultured *B. abortus* 2308 in TSB (pH 4.5), TSB (10 mM 2,2′-dipyridyl), and BMM (*Brucella* minimum medium)for 4 h or in TSB (2.5 mM H_2_O_2_) for 30 min.

### Mice and Ethics Statement

Female 4- to 6-week-old BALB/c mice were obtained from Beijing Vital River Laboratory Animal Technology Co., Ltd. All animals were handled in strict accordance with the Experimental Animal Regulation Ordinances defined by the China National Science and Technology Commission; the study was approved by the animal ethics committee of China Institute of Veterinary Drug Control.

### RNA Isolation and Reverse Transcription Polymerase Chain Reaction

We extracted the total RNA of *B. abortus* 2308 under different stress conditions and different growth stages using Bacterial RNA Kit (Omega) and reverse-transcribed into cDNA using random primers, as previously described (Liu et al., 2012). We performed RT-PCR to verify the expression of the sRNA candidates. 1 μl of cDNA sample (without dilution) or total RNA (negative control) was used as template for the PCR. The specific primers of BASI74 used for RT-PCR are listed in **Supplementary Table S1**. We analyzed the PCR products using a 2% agarose gel by electrophoresis, and the bands with the appropriate sizes were cut and sequenced by the Beijing Genomics Institute (Shenzhen, China).

### Construction of Small RNA Over-expression Strains

Each putative sRNA encoding sequence (containing the predicted sRNA sequence, about 300 nt upstream and 300 nt downstream sequences) inserted into pBBR1-MCS6 was analyzed to make sure it contains a putative promoter sequence. The constructed over-expression plasmids were verified by sequencing. For construction of sRNA over-expression strains, the pBBR1-MCS6 plasmid with putative sRNA encoding sequence was electroporated into *B. abortus* 2308, and then cells were plated onto TSA containing chloramphenicol for selection of positive clones. In addition, the over-expression strains were further verified by PCR using universal primers.

### Construction of BASI74 Deletion Mutant

Construction of recombinant plasmid and selection of marked deletion mutant were performed as previously reported (Zhang et al., 2009). The primers used to construct the recombinant plasmid were listed in **Supplementary Table S1**.

### Quantitative RT-PCR

In order to detect the expression levels of the sRNAs under different stress conditions described above, we performed RT-qPCR as previously described (Dong et al., 2013). Samples were run in triplicate and amplified in a 20 μl reaction system containing 10 μl 2 × SYBR^®^ Premix Ex TaqTM II(TAKARA), 100 nM forward and reverse primers, and 1 μl appropriately diluted cDNA sample. Primers used for RT-qPCR are provided in **Supplementary Table S1**. 16S rRNA, expression of which is relatively constant in bacteria, was used as a reference gene.

### Cellular Infections

To investigate intracellular survival of the pathogen, we evaluated the multiplication of *B. abortus* 2308 and its derived strains in J774A.1 murine macrophages. The assays were performed as previously described (Zhang et al., 2009).

### Mouse Infections

Mice were inoculated intraperitoneally with 100 μl (10^5^ CFU) of 2308-BASI74 and the parental strain *B. abortus* 2308. Five mice of one group were euthanized via carbon dioxide asphyxiation at 1 and 4 weeks post-infection. At each time point, the spleens were harvested, weighed, and then homogenized in 1 ml of peptone saline. Serial dilutions were prepared, and 100-μl aliquots of each dilution (including the undiluted organ) were plated in duplicate onto TSA plates or TSA plates with 30μg/mL chloramphenicol (Zhang et al., 2009).

### Stress Assays

We performed the stress response assays as previously reported with slight modifications as following: the *Brucella* strains derived from a single clone were grown for 48 h in 4 ml TSB medium. The bacterial cells (initial density of 1 × 10^6^ CFU/ml) were grown in BMM at 37°C with continuous shaking. The concentration of bacteria was measured every 2 days. The number of colony forming units per milliliter was obtained by plating a series of 1:10 dilutions on TSA plates.

To test if over-expression of BASI74 affected bacterial survival under acidic environments, the *Brucella* strains (with an initial density of 1 × 10^7^ CFU/ml) were cultured in TSB (pH 4.5) and the concentration of bacteria was measured at 2 h and 9 h post-inoculation.

In order to determine if over-expression of BASI74 affected bacterial survival under oxidation stress, bacterial strains were adjusted to a concentration of 1 × 10^9^ CFU/ml, and 100 μl of each bacterial strains were seeded on a TSA plate, with a 5.5 mm sterile filter paper disk in the center of each plate. We placed 10 μl of a 30% solution of H_2_O_2_ onto each disk and incubated at 37°C with 5% CO_2_. After 72 h of incubation, the zones of inhibition around each disk were measured.

In order to detect if over-expression of BASI74 affected the iron utilization, we cultured the *Brucella* strains in an iron limitation medium (TSB with 2.5, 5, and 10 mM2,2′-dipyridyl) for 48 h. The bacteria were cultured in this medium at the same initial density (1 × 10^6^ CFU/ml), and we then determined the CFUs at 48 h for each strain.

### Bioinformatics Data Analysis

To determine the position of putative promoter sequence, the upstream sequences of each verified sRNAs were analyzed using BDGP: Neural Network Promoter Prediction^1^, with the parameters for the software set at their default settings.

We predicted the target genes for the sRNA using CopraRNA^2^, with the parameters for the software set at their default settings (Wright et al., 2009).

### Verification of the Target Gene Regulated by BASI74 and β-Galactosidase Assays

The *E. coli*-based reporter system used for verification of genes regulated by BASI74 was constructed as previously described (Dong et al., 2014). The primers used to amplify BASI74 and the putative target sequences are listed in **Supplementary Table S1**, and the plasmids used in this study can be found in **Supplementary Table S2**.

### Statistical Analysis

Differences between the means of the experimental and control groups were analyzed using the independent samples *t*-test included in the program SPSS 17.0. Differences were considered significant at *p*-values of <0.05.

## Results

### Identification of Additional 43 sRNAs Expressed in *Brucella abortus* 2308

**FIGURE 1:**
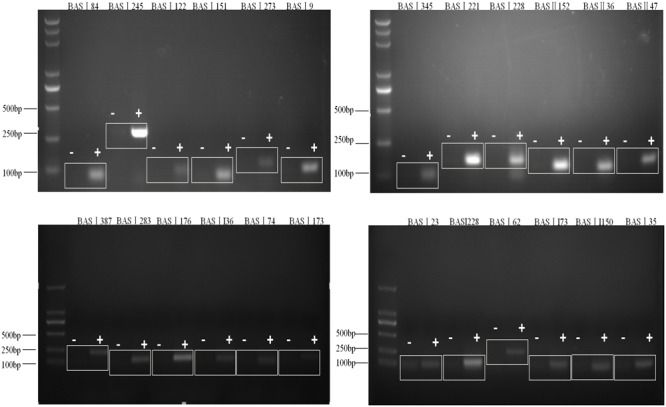
Verification of sRNAs in *B. abortus* 2308. RT-PCR verification of the transcriptional unit of sRNA candidates. RNA prepared from *B. abortus* 2308 grown to stationary phase at 37°C was used for RT-PCR. The regions to be amplified were shown by bars with numbers. “+” represents reactions with reverse transcriptase and “-” represents reactions without reverse transcriptase.

Our previous studies had identified 129 sRNAs candidates of *Brucella* using bioinformatics methods, and 7 of 20 tested sRNA candidates were verified to be present (Dong et al., 2014). In this study, we extracted the total RNA of *B. abortus* strain 2308 and detected if the remaining 109 sRNA candidates were expressed using RT-PCR. A total of 43 sRNAs could be detected by RT-PCR and sequencing (**Table 1**), out of which the RT-PCR result of 24 sRNAs were shown in **Figure 1**.

**Table 1:** Verified sRNA in this work.

sRNA Name	sRNA start	end	Length of sRNA (nt)
BAS I 365	24475	24577	102
BAS I 371	29595	29988	393
BAS I 387	47061	47307	246
BAS I 22	100793	100872	79
BAS I 23	100828	100944	116
BAS I 262	128868	129114	246
BAS I 35	254741	254855	114
BAS I 62	521937	522023	86
BAS I 74	713147	713233	86
BAS I 84	815539	815617	78
BAS I 244	1098391	1098472	81
BAS I 245	1099846	1100145	299
BAS I 122	1173218	1173318	100
BAS I 130	1221129	1221226	97
BAS I 133	1249641	1249811	170
BAS I 137	1289949	1290101	152
BAS I 151	1441442	1441521	79
BAS I 273	1445660	1445799	139
BAS I 9	1490038	1490143	105
BAS I 283	1500367	1500491	124
BAS I 176	1603445	1603565	120
BAS I 304	1648877	1649137	260
BAS I 306	1662860	1663033	173
BAS I 193	1688162	1688286	124
BAS I 214	1971684	1971765	81
BAS I 344	2005699	2006063	364
BAS I 345	2017052	2017142	90
BAS I 218	2032657	2032746	89
BAS I 221	2056362	2056509	147
BAS I 228	2084505	2084582	77
BAS II 152	873615	873740	125
BAS II 36	295058	295164	106
BAS II 47	433815	433936	121
BAS II 149	75638	75764	126
BAS II 37	309649	309775	126
BAS II 99	1099166	1099368	202
BAS II 39	325182	325397	215
BAS II 133	508838	509001	163
BAS II 5	580622	580840	218
BAS II 73	824439	824525	86
BAS II 150	824439	824613	174
BAS II 74	824439	824637	198
BAS II 117	381044	381193	149

### Identification of sRNA Over-expressed Strains With Reduced Survival Compared With Parental Strain in Macrophages

In this study, several *cis*-encoded sRNAs were verified, and it was impossible to construct mutants of *cis*-encoded sRNAs without affecting their neighboring target genes. To address this problem, we over-expressed all the 43 verified sRNAs in the wild type *Brucella* strain, and detected if the virulence of these over-expressed strains were altered.

Overall, the virulence of 42 sRNA over-expression strains were almost equivalent to that of 2308 and 2308-pBBR1, while over-expression of the sRNA BASI74(named 2308-BASI74) significantly reduced *Brucella* virulence in the macrophage infection model at 48h post-infection (*p* < 0.01) (**Table 2**).

**Table 2:** Multiplication ability of sRNA over expression strains in J774A.1 macrophages.

sRNA Name	1 h CFU	Over-expression strain/WT(1 h)	48 h CFU	Over-expression strain/WT(48 h)	sRNA Name	1 h CFU	Over-expression strain/WT(1 h)	48 h CFU	Over-expression strain/WT (48 h)
BAS I 74	1.98E + 03	33.51%	9.17E + 03	0.61%	BAS I 306	8.42E + 02	14.23%	4.98E + 05	33.40%
BAS I 22	9.67E + 02	16.34%	4.05E + 05	27.14%	BAS I 344	8.66E + 03	146.32%	8.61E + 05	57.70%
BAS I 23	8.33E + 02	14.08%	2.45E + 05	16.42%	BAS I 345	8.42E + 02	14.23%	2.28E + 05	15.30%
BAS I 35	1.28E + 03	21.55%	1.23E + 05	8.27%	BAS I 365	2.24E + 03	37.89%	4.16E + 05	27.87%
BAS I 62	2.71E + 03	45.77%	2.62E + 05	17.54%	BAS I 371	9.33E + 02	15.77%	5.83E + 05	39.04%
BAS I 9	8.33E+02	14.08%	7.52E + 05	50.38%	BAS I 387	5.42E + 03	91.55%	1.02E + 06	68.16%
BAS I 84	2.80E + 03	47.32%	4.33E + 05	29.04%	BAS II 5	2.01E + 03	33.94%	3.66E + 05	24.52%
BAS I 122	1.10E + 03	18.59%	1.15E + 06	77.08%	BAS II 36	8.42E + 02	14.23%	3.18E + 05	21.34%
BAS I 130	8.67E + 03	146.48%	1.10E + 06	73.39%	BAS II 37	1.15E + 03	19.44%	6.55E + 05	43.90%
BAS I 133	1.63E + 03	27.46%	5.52E + 05	36.97%	BAS II 39	1.57E + 03	26.48%	3.68E + 05	24.69%
BAS I 137	3.11E + 03	52.53%	8.50E + 05	56.97%	BAS II 47	1.99E + 03	33.67%	4.15E + 05	27.81%
BAS I 151	1.91E + 03	32.25%	7.80E + 05	52.28%	BAS II 73	9.08E + 02	15.35%	3.22E + 05	21.56%
BAS I 176	2.28E + 03	38.45%	2.84E + 05	19.05%	BAS II 74	1.15E + 03	19.49%	6.33E + 05	42.45%
BAS I 193	1.49E + 03	25.22%	5.88E + 05	39.43%	BAS II 99	1.60E + 03	27.04%	3.33E + 05	22.34%
BAS I 214	2.31E+03	39.01%	5.47E + 05	36.64%	BAS II 117	5.42E + 03	91.55%	6.23E + 05	41.78%
BAS I 218	1.79E + 03	30.29%	5.58E + 05	37.42%	BAS II 133	3.27E + 03	55.21%	9.51E + 05	63.73%
BAS I 221	8.33E + 03	140.83%	1.25E + 06	83.44%	BAS II 149	1.50E + 03	25.35%	4.96E + 05	33.23%
BAS I 228	1.81E + 03	30.56%	1.00E + 06	67.02%	BAS II 152	2.90E + 03	49.01%	8.02E + 05	53.73%
BAS I 244	5.50E + 03	92.95%	9.47E + 05	63.45%	BAS II 150	1.50E + 03	25.35%	4.96E + 05	33.23%
BAS I 245	1.03E + 03	17.46%	8.27E + 05	55.41%	2308-pBBR1	2.14E + 03	36.20%	1.00E + 06	67.09%
BAS I 262	9.25E + 02	15.63%	3.97E + 05	26.59%	2308	5.92E + 03	100.00%	1.49E + 06	100.00%
BAS I 273	8.42E + 02	14.23%	3.95E + 05	26.47%					
BAS I 283	7.08E + 03	119.71%	5.17E + 05	34.63%					
BAS I 304	1.28E + 03	21.68%	4.19E + 05	28.09%					

### Over-expression of BASI74 Affected the Virulence of *B. abortus* 2308

To further confirm the relationship between BASI74 and reduced survival ability in macrophages, a BASI74-deletion strain(named ΔBASI74) was constructed and the virulence of 2308-BASI74 and ΔBASI74 in J774A.1 macrophages was detected at different time points.

Before the macrophage infection assay, the expression of BASI74 was detected in both 2308-BASI74 and ΔBASI74. The results of RT-qPCR showed that the expression levels of BASI74 were not significantly different between 2308 and ΔBASI74, while that of 2308-BASI74 was about 8-fold higher than that of 2308(**Supplementary Table S3**). According to the blast result of BASI74 sequence in *B. abortus* 2308, several highly homologous sequences were found in both chromosomes, I and II (**Supplementary Table S4**).

**FIGURE 2:**
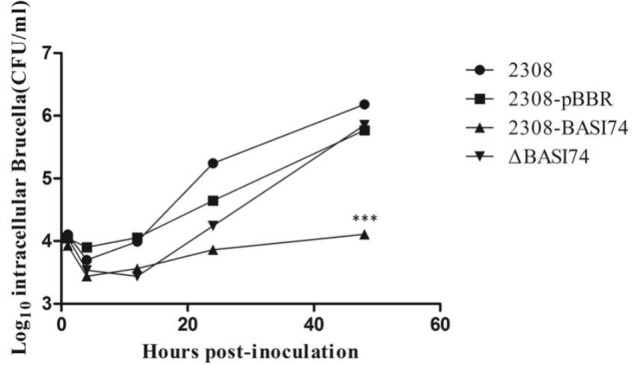
Effect of BASI74 on *Brucella* virulence in macrophage infection model. Multiplication of *B. abortus* 2308, 2308-BASI74, ΔBASI74, and 2308-pBBR1 in J774A.1 macrophages over 48 h. Values represent the means of three independent experiments performed in duplicate, and error bars indicate the SD. ^∗∗∗^ indicated the *p* < 0.001.

As shown in **Figure 2**, the intracellular bacteria load of 2308-BASI74 was significantly reduced at 48 h post-infection compared to that of 2308 and 2308-PBBR1 (*p* < 0.01). However, the survival ratio of ΔBASI74 showed no difference, compared with that of 2308 at each time point.

**FIGURE 3:**
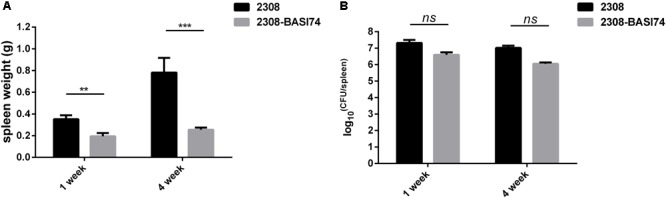
Effect of BASI74 on *Brucella* virulence in mice infection model. **(A)** The spleen weight of 2308-BASI74 and 2308 infected mice at 1 and 4 weeks post infection. **(B)** The splenic CFUs of 2308-BASI74 and 2308 in infected mice at 1 and 4 weeks post infection. ^∗∗^ indicated the *p* < 0.01. ^∗∗∗^ indicated the *p* < 0.001.

To evaluate the virulence *in vivo*, BALB/c mice were infected with both 2308-BASI 74 and *B. abortus* 2308. Compared with the parental strain 2308, the spleen weight of 2308-BASI74 infected mice was significantly lighter at both 1 week(*p* < 0.01) and 4 weeks(*p* < 0.001) post-infection (**Figure 3A**), while no significant difference was observed in the splenic CFUs between 2308-BASI74 and 2308-infected groups at each time point(*p* > 0.05) (**Figure 3B**).

### Expression Pattern of BASI74 in *B. abortus* 2308

**FIGURE 4:**
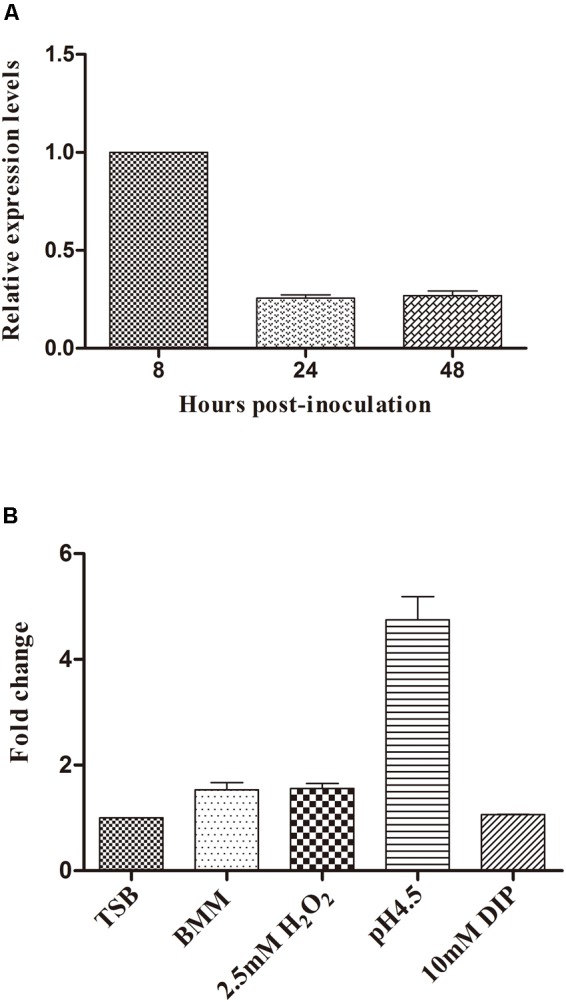
Expression pattern of BASI74 in *B. abortus* 2308. **(A)** Expression levels of BASI74 at different growth stages in TSB medium. **(B)** Expression levels of BASI74 under different stress conditions. *B. abortus* 2308 from a single clone was grown for 48 h in TSB medium and then cultured in TSB (pH 4.5), TSB + 10 mM 2,2′-dipyridyl, BMM (*Brucella* minimum medium) for 4 h or in TSB + 2.5 mM H_2_O_2_ for 30 min. The expression levels of BASI74 under different stress conditions were compared to samples cultured in TSB medium. DIP stands for 2,2′-dipyridyl.

RT-qPCR with RNA samples isolated from the bacteria grown under different stress conditions or harvested at different stages was performed in order to characterize the expression pattern of BASI74. We found that BASI74 was produced at all growth phases, and the expression level increased to the peak at 8 h post-incubation (**Figure 4A**). The expression levels of BASI74 were not significantly changed under iron deficiency (10 mM 2,2′-dipyridyl for 4 h) or oxidative (2.5 mM H_2_O_2_ for 30 min) stress, or even in BMM compared with in normal TSB culture. However the level of BASI74 increased more than 4-fold under acidic (pH 4.5 for 4 h) stress than in normal TSB control (**Figure 4B**).

### The BASI74 Was Involved in Stress Responses

The characteristics of 2308-BASI74 in macrophages promoted us to study the underlying mechanisms. Previous studies have demonstrated that many sRNAs are related to stress response, and therefore the survival ability of the over-expression strains under different stress conditions was tested.

**FIGURE 5:**
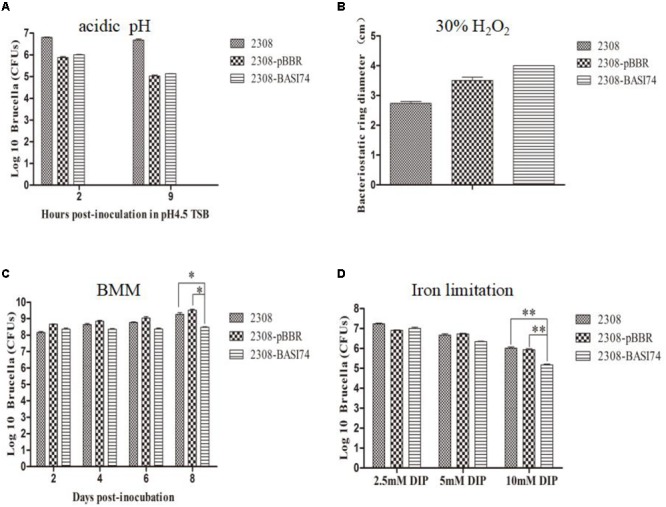
Growth behavior of strains over-producing BASI74 under different stress conditions. **(A)** Over-expression of BASI74 did not affect the survival ratio of *B. abortus* in acidic medium (TSB pH 4.5). **(B)** The 2308-BASI74 strain had equal sensitivity to 30% H_2_O_2_ compared to two control strains. **(C)** BASI74 affected bacteria growth in *Brucella* minimum medium. **(D)** Over-expression of BASI74 reduced the survival ratio of *B. abortus* under iron-limiting condition. Values represent the means of three independent experiments, and error bars indicate the SD. ^∗^ indicated the *p* < 0.05. ^∗∗^ indicated the *p* < 0.01.

As shown in **Figure 5A**, the survival ratio of 2308-BASI74 cultured in an acidic medium for 9 h was almost the same as that of 2308-pBBR1 and 2308. Neither did we find significant differences of growth ratio among these three strains in the H_2_O_2_ disk sensitivity assays (**Figure 5B**). In BMM culture, the growth ratio of 2308-BASI74 gradually deviated since 4 days post-incubation compared with that of 2308 and 2308-pBBR1 cells, and turned out to be significantly lower at 8 days post-incubation (*p* < 0.05) (**Figure 5C**). In addition, the survival ratio of the 2308-BASI74 was much lower than that of 2308 and 2308-pBBR1 when cultured in iron limited TSB (10 mM 2,2′-dipyridyl) for 48 h (**Figure 5D**). These data revealed that BASI74 was involved in growth in iron-limiting medium and BMM.

### Identification of Targets Regulated by BASI74

To identify the genes regulated by the BASI74 RNA, we performed an *in silico* analysis with sTarPicker (see footnote 2).

As shown in **Table 3**, for BAB1_1361, BAB1_1335, the β-galactosidase activity of the strains containing the combination of the sRNA-encoding plasmid and target *lacZ* fusion plasmids were significantly reduced compared with the vector and *lacZ* fusion plasmids combination group. On the contrary, co-expression of BASI74 with the 5’-UTR of BAB1_1154 or BAB1_0847 *lacZ* fusion plasmids significantly increased the β-galactosidase activity. For BAB1_0097 and BAB1_0343, no obvious difference was observed between the BASI74 and vector group.. Except for that of BAB1_1154 (encoding cytosine-N4-specific DNA methyltransferase), functions of all other three targets were still unknown.

**Table 3:** Verification of the interaction between BASI74 and putative target sequences.

Putative target genes	β- galactosidase activity (Miller units)		Fold change BASI74/vector	*p*-value
	pUT18C	pUT18C-BASI74		
BAB1_0097	9.21 ± 2.23	11.61 ± 2.71	1.26	0.8093
BAB1_0343	3.15 ± 1.21	2.01 ± 0.83	0.64	0.1253
BAB1_0847	15.57 ± 2.77	23.65 ± 2.57	1.52	<0.05
BAB1_1154	14.99 ± 2.41	47.18 ± 1.935	3.15	<0.001
BAB1_1335	4.18 ± 0.32	1.36 ± 0.48	0.33	<0.05
BAB1_1361	5.48 ± 1.81	2.03 ± 1.64	0.37	<0.05

*The data were expressed as averages ± standard deviations (SD). Three independent experiments were performed.*

To further determine whether these targets were regulated by BASI74, the expression level of four putative targets was tested by RT-qPCR in both ΔBASI74 and 2308-BASI74. As shown in **Table 4**, the transcriptional level of all four verified targets was upregulated in 2308-BASI74, while none of the four targets was affected in the ΔBASI 74.

**Table 4:** The transcriptional levels of four verified target genes in 2308-BASI74 and ΔBASI74.

Gene	Fold change	
	2308-BASI74 vs. 2308	ΔBASI74 vs. 2308
BAB1_1361	4.52	1.32
BAB1_1335	2.69	1.17
BAB1_1154	7.89	0.93
BAB1_0847	3.89	0.77

## Discussion

Previous studies have demonstrated that sRNAs were related with the proper expression of virulence factors in a variety of pathogenic bacteria (Papenfort and Vogel, 2010), and several recent studies also showed that sRNAs directly correlated with the virulence of organisms such as *Listeria* (Mraheil et al., 2011), *Salmonella* (Gong et al., 2011), *Vibrio* (Song et al., 2008), *Yersinia* (Koo et al., 2011), and *Brucella* (Caswell et al., 2012).

In this study, it was interesting to find that over-expression of BASI74 locus reduced *Brucella* virulence in macrophages, while deletion of putative BASI74 encoding sequence did not affect *Brucella* virulence. The results of RT-qPCR showed that the transcriptional level of BASI74 between 2308 and ΔBASI74 were not significantly changed, while that of 2308-BASI74 was about 8-fold higher than that of 2308, which might possibly explain the difference of virulence between 2308-BASI74 andΔBASI74. We speculated that there possibly existed more than one locus encoding BASI74 in the genome of 2308.

Further, we observed a consistent trend in the downstream target genes. The transcriptional level of four verified target genes were changed more than 2-fold in 2308-BASI74, while none of the four targets was affected in ΔBASI74.

In a previous study, it was also demonstrated that over-expression of sRNAs could result in more dramatic effects on their regulated targets than sRNAs deletion (Koo et al., 2011). Our result was consistent with data previously reported.

Taken together, these data indicated that the reason that ΔBASI74 could not significantly affect the virulence of *Brucella* strains might be explained by the redundancy in genetic structure and function.

Previous studies demonstrated that AbcR sRNAs had redundant and compensatory functions in *B. abortus* 2308 (Caswell et al., 2012). In addition, the four Qrr sRNAs involved in the regulation of quorum sensing are redundant in *Vibrio cholerae* (Lenz et al., 2004). In our study, the probable multiple copies of BASI74 might indicate the important role of this sRNA, and the redundancy of this sRNA may be an evolutionary adaption ensuring the proper expression of essential genes.

As a facultative intracellular pathogen, *B. abortus* encounters formidable environmental stresses such as nutrient deprivation during its interactions with the host cells (Roop et al., 2009). In addition, *Brucella* strains required iron transporters for the expression of wild type virulence in natural and experimental hosts (Roop, 2012). Our results that 2308-BASI74 exhibited lower growth in iron-limiting and nutrient deprivation medium indicated that the attenuation of 2308-BASI74 was probably related with its reduced tolerance under these two types of stresses.

Although 2308-BASI74 was attenuated in macrophage infection model, no significant difference of virulence was observed in the mice infection assay at different time points (**Figure 3B**). This disagreement of *Brucella* virulence tested by macrophage and mice infection models was not uncommon. In the study of *Brucella* quorum sensing regulator BlxR, the Δ*blxR* strain exhibited reduced growth in macrophages, while this mutant was not highly attenuated in mice (Rambow-Larsen et al., 2008). Besides, it was worthy noting that the spleen weight of the mice infected with 2308-BASI74 was significantly lighter than that of 2308 in mice infection models at both 1 and 4 weeks post-infection. This observation, which was also found in the mice infection assay of *Brucella* attenuated strain, indicated that the 2308-BASI74 might induce a different immune response in the mice infection model.

Previous studies have demonstrated that bacterial DNA methyltransferases were not only associated with restriction-modification systems, but also with chromosome replication, transcription, repair, and many other fundamental processes (Reisenauer et al., 1999). In addition, some recent studies have demonstrated that DNA adenine methylation play an important role in host-pathogen interactions (Marinus and Casadesus, 2009). In *B. abortus*, the CcrM DNA methyltransferase was also reported to be essential for viability, and its over-expression attenuated intracellular replication in murine macrophages (Robertson et al., 2000). In *Helicobacter pylori*, C5-cytosine methylation also affects the expression of several genes related to motility, adhesion, and virulence (Kumar et al., 2012). However, the cytosine-N4-specific DNA methyltransferase can hardly be related with bacterial virulence. As a *trans*-encoded sRNA, BASI74 could regulate more than one target mRNA. In this study, we only verified the top six putative targets with the highest scores in the prediction result, and more targets of BASI74 needs to be verified in the future. Thus, we hypothesized that over-expression of BASI74 might have various effects on more different targets including the target gene BAB1_1154 encoding DNA methylation.

## Author Contributions

HD wrote the paper. XP, YL, TW, and XW performed the experiments. CW and QW conceived and designed the experiments. YD, TH, LY, and JD analyzed the data.

## Conflict of Interest Statement

The authors declare that the research was conducted in the absence of any commercial or financial relationships that could be construed as a potential conflict of interest.

## Footnotes

1 http://www.fruitfly.org/seq_tools/promoter.html

2 http://rna.informatik.uni-freiburg.de
