# Supplementary material for: BASI74, a Virulence-Related sRNA in Brucella abortus
Source: Front Microbiol. 2018 Sep 13;9:2173. doi: 10.3389/fmicb.2018.02173 (PMC6146029; doi:10.3389/fmicb.2018.02173)
Supplement: Supplementary file 1 [file Table_1.DOCX]

Table S1 Primers used in this study

| Primers | Sequences (5’to 3’) | Used for |
| --- | --- | --- |
| P1361F | CCCAAGCTTCCCGTCCGGCCCGAACCTG | Construct target *lacZ* fusion plasmids |
| P1361R | GGGGTACCATGAACATTTTCGAGCCAAAA |  |
| P343F | CCCAAGCTTGGCTGCTCTCTAGAGCGGT |  |
| P343R | GGGGTACCATTATTCACTCTCCTTCCAATC |  |
| P1335F | CCCAAGCTTCCATATTGCCCTCAAAAAAT |  |
| P1335R | GGGGTACCATCTGAACGGCGTGCGCTGTAC |  |
| P847F | CCCAAGCTTATTTGTGTCGTAACTTGCGGA |  |
| P847R | GGGGTACCATGCCCCGTTTCCTTCTGGA |  |
| P1154F | CCCAAGCTTTGTTTAAGTTATCTAGAGC |  |
| P1154R | GGGGTACCATGCAACTCACCCCCAGAGT |  |
| P97F | CCCAAGCTTTTTCAAACCTTTTAGGA |  |
| P97R | GGGGTACCATGATTGCTGAAGCTGGCGT |  |
| BASI74F | GGGGTACC CGATACGAAAATCTGGA | Construct high-copy sRNA expression plasmids |
| BASI74R | CGGGATCC GCGCATCCGCCAATGC |  |
| 16S rRNAf | TACCAGCCCTTGACATCC | RT-qPCR |
| 16S rRNAr | TCATCCCCACCTTCCTCT |  |
| BASI74-1 | CGCTAAAGCATTTTCGAGCC |  |
| BASI74-2 | CGCATCGAAGCGGGATCA |  |
| BAB1_0847f | TGCCTTTGGTTTCACTGCC |  |
| BAB1_0847r | AGATGCCTGCTGGTCTTTCA |  |
| BAB1_1154 f | CGAACTGGCGGAAGAGGCT |  |
| BAB1_1154 r | ATGCACGATGAATACACTTGACGA |  |
| BAB1_1335f | AGCGGGATCAGAAATCAGTC |  |
| BAB1_1335r | TGCGTAGGATAATGCGTAAAA |  |
| BAB1_1361 f | GGGCTCACGGCAAATGAG |  |
| BAB1_1361 r | ACGGTCGAAGCTGCTGGA |  |
| D-BASI74F1 | CCGCTCGAGCATTATCTCAACGGTTTC |  |
| D-BASI74F2 | CGGTTAAGGATCCTCACGCTCTTGCCGGATA | Construct recombine plasmids |
| D-BASI74R1 | GGATCCTTAACCGGCGTATTTCAGG |  |
| D-BASI74R2 | GCTCTAGAATGTCTCTCCGTTTTTCGACTTC |  |
