# Supplementary material for: BASI74, a Virulence-Related sRNA in Brucella abortus
Source: Front Microbiol. 2018 Sep 13;9:2173. doi: 10.3389/fmicb.2018.02173 (PMC6146029; doi:10.3389/fmicb.2018.02173)
Supplement: Supplementary file 2 [file Table_2.docx]

**Table S2 Bacteria strains and plasmids used in this study**

| Bacteria or Plasmid name | Characteristics | References  or source |
| --- | --- | --- |
| *B. abortus* 2308 | Wild-type, smooth, virulent | Laboratory stock |
| *E. coli*  DH5α | F^−^ φ80d*lacZ*ΔM15 Δ(*lacZYA*-*argF*)*U169 recA1 endA1 hsdR17*(r_K_^−^m_K_^+^) *supE44 thi-1 gyrA relA1* | Invitrogen |
| Plasmids |  |  |
| pBBR1-MCS6 | Broad-host-range plasmid, Cm^r^ | Kovach et al., 1994 |
| pMR10 | RK2 derivative, Low-copy plasmid, Kan^r^ | R. Wright |
| pUT18C | Small RNA expression plasmid, *cyaA*T18 fusion; Amp^r^ |  |
| pUT18C-BASI74 | pUT18C derivative with BASI74 inserted in *Bam*HI*&Kpn*I site, Amp^r^ | This study |
| pMR-*lacZ* | pMR10 derivative with *lacZ* ORF inserted in *Hin*dⅢ*&Bam*HⅠsite, Kan^r^ | This study |
| pMR-*lacZ*0097 | pMR-*lacZ* derivative with predicted target sequence(BAB1_0097) of BASI74 inserted in *Hin*dⅢ*&Kpn*Ⅰ, Kan^r^ | This study |
| pMR-*lacZ*0343 | pMR-*lacZ* derivative with predicted target sequence(BAB1_0343) of BASI74 inserted in *Hin*dⅢ*&Kpn*Ⅰ, Kan^r^ | This study |
| pMR-*lacZ*0847 | pMR-*lacZ* derivative with predicted target sequence(BAB1_0847) of BASI74 inserted in *Hin*dⅢ*&Kpn*Ⅰ, Kan^r^ | This study |
| pMR-*lacZ*1154 | pMR-*lacZ* derivative with predicted target sequence(BAB1_1154) of BASI74 inserted in *Hin*dⅢ*&Kpn*Ⅰ, Kan^r^ | This study |
| pMR-*lacZ*1335 | pMR-*lacZ* derivative with predicted target sequence(BAB1_1335) of BASI74 inserted in *Hin*dⅢ*&Kpn*Ⅰ, Kan^r^ | This study |
| pMR-*lacZ*1361 | pMR-*lacZ* derivative with predicted target sequence(BAB1_1361) of BASI74 inserted in *Hin*dⅢ*&Kpn*Ⅰ, Kan^r^ | This study |
