# Supplementary material for: BASI74, a Virulence-Related sRNA in Brucella abortus
Source: Front Microbiol. 2018 Sep 13;9:2173. doi: 10.3389/fmicb.2018.02173 (PMC6146029; doi:10.3389/fmicb.2018.02173)
Supplement: Supplementary file 3 [file Table_3.docx]

**Table S3 The transcriptional levels of BASI74 in 2308-BASI74, ΔBASI74 and 2308-pBBR1.**

| *Brucella* strain | Fold change  (*Brucella* strain vs 2308) |
| --- | --- |
| 2308-BASI74 | 8.32 |
| ΔBASI74 | 0.81 |
| 2308-pBBR1 | 1.12 |
