# Supplementary material for: BASI74, a Virulence-Related sRNA in Brucella abortus
Source: Front Microbiol. 2018 Sep 13;9:2173. doi: 10.3389/fmicb.2018.02173 (PMC6146029; doi:10.3389/fmicb.2018.02173)
Supplement: Supplementary file 4 [file Table_4.docx]

**Table S4 The top 10 homologous sequences of BASI74 in the genome of *Brucella abortus* 2308.**

|  | Sequence start | Sequence end | Chromosome | length | Identity |
| --- | --- | --- | --- | --- | --- |
| 1 | 1173234 | 1173318 | I | 85 | 84/85(99%) |
| 2 | 1441437 | 1441520 | I | 84 | 83/84(99%) |
| 3 | 1316961 | 1317039 | I | 79 | 78/79(99%) |
| 4 | 214475 | 214559 | II | 85 | 84/85(99%) |
| 5 | 2084499 | 2084582 | I | 86 | 83/86(97%) |
| 6 | 408256 | 408333 | I | 78 | 77/78(99%) |
| 7 | 369966 | 370050 | I | 85 | 81/85(95%) |
| 8 | 100786 | 100869 | I | 84 | 80/84(95%) |
| 9 | 100786 | 100869 | I | 84 | 80/84(95%) |
| 10 | 1032795 | 1032874 | I | 80 | 76/80(95%) |
